# Simulation Prediction and Experimental Research on Surface Morphology of Ball Head Milling Processing

**DOI:** 10.3390/ma18102355

**Published:** 2025-05-19

**Authors:** Youzheng Cui, Xinmiao Li, Minli Zheng, Haijing Mu, Chengxin Liu, Dongyang Wang, Bingyang Yan, Qingwei Li, Hui Jiang, Fengjuan Wang, Qingming Hu

**Affiliations:** 1 School of Mechanical and Electronic Engineering, Qiqihar University, Qiqihar 161006, China; 18646407893@163.com (X.L.); muhaijinghust@163.com (H.M.); liuchengxin1088@163.com (C.L.); 19190706213@163.com (D.W.); 18932293030@163.com (B.Y.); 18814663144@163.com (Q.L.); qzsk_jh@163.com (H.J.); 02742@qqhru.edu.cn (F.W.); 02510@qqhru.edu.cn (Q.H.); 2 Key Laboratory of Advanced Manufacturing and Intelligent Technology, Ministry of Education, Harbin University of Science and Technology, Harbin 150080, China; minli@hrbust.edu.cn; 3 QiQiHar Heavy CNC Equipment Co., Ltd., Qiqihar 161005, China; 4 The Engineering Technology Research Center for Precision Manufacturing Equipment and Industrial Perception of Heilongjiang Province, Qiqihar 161006, China; 5 The Collaborative Innovation Center for Intelligent Manufacturing Equipment Industrialization, Qiqihar 161006, China; 6 School of Mechatronics Engineering, Harbin Institute of Technology, Harbin 150001, China

**Keywords:** surface morphology simulation, ball-end milling, Cr12MoV, Z-map algorithm, tool vibration

## Abstract

With the aim of improving the machined surface quality of die steel, this paper takes Cr12MoV quenched die steel as the research object and proposes a ball head milling surface morphology prediction model that comprehensively considers influencing factors, including tool vibration, eccentricity, as well as deformation. By setting key parameters, such as line spacing, feed per tooth, cutting depth, and phase difference, the system analyzed the influence of each parameter on the residual height and surface roughness of the machined surface. High-speed milling experiments were conducted, and the surface morphology of the samples was observed and measured under a microscope. The simulation results show good agreement with the experimental data, with errors within 7%~15%, proving the accuracy of the model. This study can provide theoretical support and methodological guidance for surface quality control and processing parameter optimization in complex mold surface machining.

## 1. Introduction

Quenched steel is known for its high hardness, wear resistance, and ease of machining, making it extensively applied in industries, like aerospace, construction machinery, and automotive manufacturing [1,2]. In the automotive industry, quenched die steel is one of the indispensable processing tools. The surface condition after high-speed milling is crucial for the study of surface integrity and is also a key factor in ensuring component performance [3]. Moreover, it exerts a direct influence on the operational lifespan of the mold. Therefore, it is particularly crucial to delve into the morphological characteristics of the processed surface in order to accurately grasp its mechanism of action and make precise predictions of surface morphology. This not only helps to improve the accuracy of mold processing, but also effectively reduces production costs, which has important theoretical value and practical guidance significance.

In recent years, significant progress has been made in the research of three-dimensional surface morphology simulation, which has significantly improved the surface quality and prediction accuracy of complex curved surface part machining. Quinsat et al. [4,5] introduced a 3D surface morphology simulation model that considers tool rotation and translation during machining, yielding more accurate results; their model also allows machining parameters to be adjusted based on the predicted surface roughness. Zhuo, Y. [6] and others have developed a new prediction method that takes into account the vibration and material removal effects in the course of cutting. This method is particularly suitable for modeling and testing the surface morphology of thin-walled parts during peripheral milling. Experiments have shown that it has good accuracy and practicality. Urbikain, G. [7] and others combined geometric modeling and empirical modeling to predict the surface roughness when machining free-form surfaces using a new barrel end mill. They also included the factor of tool runout. By using geometric models based on tool travel paths and mathematical models based on cutting conditions, these models have been validated through experiments on Al7075T6 aluminum alloy and Ti6Al4V titanium alloy, demonstrating high-precision predictive ability. The error of geometric models usually does not exceed 15%, while the error of empirical models does not exceed 10%, among which the feed rate exhibits a notably pronounced effect on the resulting surface roughness. This study provides a reliable tool for efficient and precise machining of complex curved parts. Buj-Corral et al. [8] constructed a numerical model based on the principle of intersection between geometric cutting tools and workpieces, investigating the influence of feed rate and radial depth of cut on the surface topography generated during ball-end milling operations. The results showed that lower surface roughness and shorter processing time can be achieved simultaneously at smaller cutting depths and higher feed rates, which helps to reduce milling and polishing costs and improve processing efficiency and surface quality. Zhang Xiangyu et al. [9] employed an orthogonal experimental design to examine how various process parameters (spindle speed, feed rate, milling depth, milling width) influence the surface roughness of a tin-bismuth alloy. They identified an optimal parameter combination that reduced surface roughness to 0.233 μm, markedly improving the machined surface quality. Wang et al. proposed a method to control surface roughness in five-axis milling through surface morphology analysis. A predictive model was constructed to simulate surface topography based on an elliptical tool trajectory, with a focused examination of how tool runout and workpiece curvature impact the resultant surface characteristics. By solving nonlinear over-determined equations, they optimized the feed rate to ensure a uniform roughness distribution at various positions on the workpiece. This approach improves the uniformity and quality of surface finish while maintaining steady machining efficiency. Wang, L. [10] et al. devised a technique to regulate surface roughness in five-axis milling by analyzing surface features. Researchers worldwide have carried out extensive studies concentrating on modeling and simulating milled surface topography, with the aim of accurately analyzing machined surfaces and effectively controlling their quality. Zhang Wei et al. [11] constructed a model for secondary machining of surface texture using ellipsoidal milling cutters and explored how various milling parameters affect surface texture characteristics. The accuracy of texture simulation was verified through milling experiments. Juan Wei et al. [12] developed a model to predict surface characteristics in ball-end milling using homogeneous coordinate transformation matrices, examining how different parameters influence the resulting surface morphology. Sylvain et al. [13] developed a model for predicting milling surfaces using N-buffer technology, which can take into account local fluctuations in feed rate and is suitable for predicting surface features of free-form surfaces. Gao et al. [14] applied a Z-map algorithm alongside milling experiments to study how cutting impact and tool wear affect surface morphology. They found the simulated data closely matched experimental results, confirming the prediction model’s accuracy. This study establishes a theoretical framework to support the optimal selection of machining parameters. Lei Zhang et al. [15] studied the influence and prediction of tool wear on the surface roughness of workpieces. The tool wear area and wear position angle have a significant impact on the surface roughness of workpieces. When the wear area and position angle are controlled within a reasonable range, it actually helps to improve the surface quality of workpieces. Dong et al. developed an improved Z-map algorithm that does not require segmentation of cutting tools for simulating milling surfaces and confirmed its effectiveness and high consistency with simulated surface features through testing [16]. Lazoglu et al. proposed an analytical model for ball-end milling, integrating key machining parameters—including feed rate, flute count, step-over distance, and cutting depth—to predict surface topography and roughness outcomes [17]. Felh ő et al. carried out experimental studies on surface roughness modeling in face milling by systematically adjusting process parameters, including feed rate, cutting speed, and axial depth of cut. A predictive model was established based on response surface methodology, which enabled precise prediction of the surface roughness after milling and clarified the individual effects of each cutting parameter on the surface roughness characteristics [18]. Scholars, such as Yu Yongquan, have explored the surface characteristics of non-circular gears and found that the determining factors for the remaining height are the feed interval and feed rate per turn [19]. Xu Jin et al. [20] carried out a comprehensive investigation on the mechanism underlying surface texture formation during the milling of ultra-high-strength steel AF1410. The study also pointed out how geometric elements and dynamic deformation jointly affect surface roughness in milling operations, and the error range of simulation results was controlled within 10.9%, thus verifying the practicality of the model. Li Yuan et al. explored the principle of surface forming at different positive angles during nanosecond laser milling of aluminum alloys [21]. At the same time, Lu Xiaohong and her team [22] constructed a model to simulate surface morphology. After actual milling experiments, the model demonstrated high accuracy.

Through a comprehensive review of the existing literature, it is found that most of the existing surface topography simulation models are based on idealized assumptions, which often simplify the dynamic behavior of the tool and ignore the influence of important factors, such as tool vibration, eccentricity error, and deformation. Although Cr12MoV die steel is widely used in die and mold processing because of its high hardness and good wear resistance, there is still little research on the evolution of surface morphology, and there is a lack of a three-dimensional surface morphology prediction model that can comprehensively consider the dynamic influence of multiple factors. In order to make up for the above shortcomings, a surface morphology prediction model suitable for Cr12MoV die steel ball end milling was constructed. The proposed model incorporates factors, such as tool vibration, spindle eccentricity, and tool deformation. Based on the cutting edge trajectory modeling and Z-map algorithm, the simulation and prediction of 3D surface topography were implemented using a MATLAB (MathWorks, Natick, MA, USA, Version R2023a) platform. This method provides an effective theoretical basis and reference for optimizing processing parameters and reducing processing costs.

## 2. Materials and Methods

### 2.1. Establishing a Mathematical Model for the Cutting Edge in Ball-Head Milling

**Figure 1:**
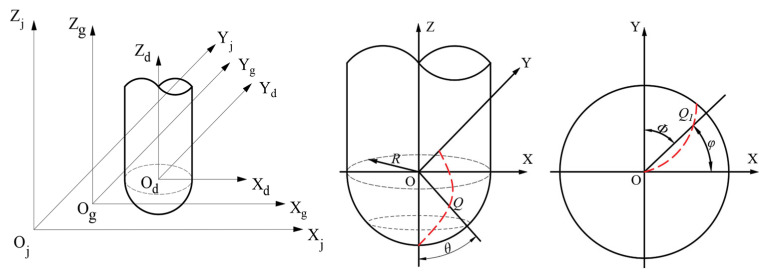
Geometric coordinate model of ball end milling cutter.

A precise description of the milling motion is the core requirement for developing a surface morphology simulation model in ball-end milling. For this purpose, it is critical to establish the trajectory equation of the cutting edge of the ball-end mill. Such an equation accurately describes the motion track of each discrete point along the cutting edge during milling. In this work, the center of the ball-end cutter is defined as the origin of the coordinate system, since the ball-end milling cutter contacts the workpiece through its cutting edges. As illustrated in Figure 1, Point *Q* is any point on the cutting edge. The distance from point *Q* to the coordinate system origin *O* is the radius ***R*** of the ball head. The included angle between the straight line *OQ* and axis *Z* is represented by ***θ***. In the figure, after any point *Q* of the cutting edge of the tool is projected to the XY plane, the included angle between the projection point *Q*_1_ and point *O* and axis *X* is ***φ***, the *Z*-axis is consistent with the cutter’s axial direction. The cutting edge is divided into *n* discrete points, and the coordinates of each point *Q* are expressed as (***x***, ***y***, ***z***).

(1) $$\left[\begin{array}{c} x\\ y\\ z \end{array}\right]=\left[\begin{array}{c} R\sin\theta \cos\left(\tan\beta \ln\left(\cot\left(\theta /2\right)\right)\right)\\ R\sin\theta \sin\left(\tan\beta \ln\left(\cot\left(\theta /2\right)\right)\right)\\ -R\cos\theta \end{array}\right]$$

In the equation, (***x***, ***y***, ***z***) represents the coordinates of point *Q* at any location within the tool coordinate system, ***θ*** denotes the angular position of *Q* along the cutting edge, and ***R*** refers to the radius of the ball-end portion of the ball-end milling cutter.

The angle formed between the line connecting the projection point *Q*_1_ to the origin *O* and the positive direction of the *Y*-axis is defined as the phase angle. If the tool has *z* cutting edges, the phase angle corresponding to the *i*-th cutting edge can be described by Equation (2):

(2) $$\varphi_{i}=\varphi_{1}+2\pi \left(i-1\right)/z$$

In this study, we define the phase difference (Δ*φ*) as the angular offset between adjacent cutter teeth. This parameter is crucial for modeling the overlapping trajectory and resulting surface morphology during the ball-end milling process. The angular spacing between adjacent cutting edges of a ball-end milling cutter is evenly arranged. By rotating the first cutting edge through an angle ***φ_i_***, the position of the *i*-th cutting edge can be determined. Consequently, the coordinates of any point *Q* located on the *i*-th cutting edge during the machining process can be formulated as:

(3) $$\left[\begin{array}{c} x_{i}\\ y_{i}\\ z_{i}\\ 1 \end{array}\right]=\left[\begin{array}{cccc} \cos\left(\varphi_{i}-\varphi_{1}\right) & -\sin\left(\varphi_{i}-\varphi_{1}\right) & 0 & 0\\ \sin\left(\varphi_{i}-\varphi_{1}\right) & \cos\left(\varphi_{i}-\varphi_{1}\right) & 0 & 0\\ 0 & 0 & 1 & 0\\ 0 & 0 & 0 & 1 \end{array}\right]$$

In the formula, (***x_i_***, ***y_i_***, ***z_i_***)—*Q* is the coordinate of the first cutting edge.

**Figure 2:**
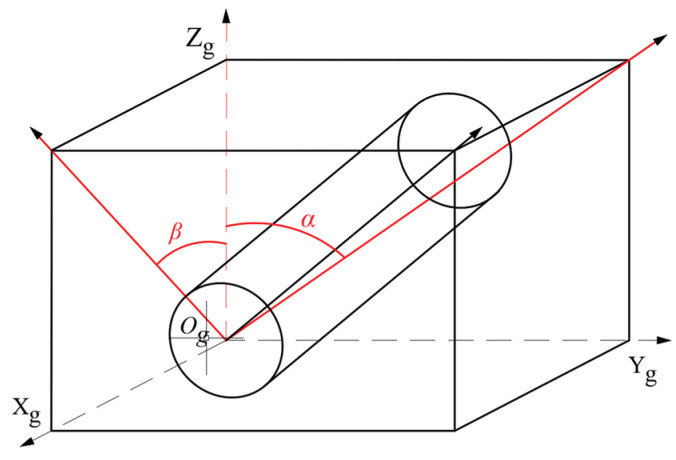
Machining inclination angle of ball nose milling cutter.

During the milling process, the tool tip does not actually participate in cutting but instead squeezes the workpiece [23]. Therefore, in the actual processing, it is often necessary to determine the appropriate processing angle according to the specific requirements. For the convenience of research, In this paper, the machining inclination angle is divided into a lateral inclination angle ***α*** and a forward inclination angle ***β***, as illustrated in Figure 2. Introduce angle transformation matrices as *M*_1_ and *M*_2_.

By reasonable angle transformation, the transformation matrices of sideslip angle and forward tilt angle can be obtained as follows:

(4) $$M_{1}=\left[\begin{array}{cccc} 1 & 0 & 0 & 0\\ 0 & \cos\alpha & -\sin\alpha & 0\\ 0 & \sin\alpha & \cos\alpha & 0\\ 0 & 0 & 0 & 1 \end{array}\right]$$

(5) $$M_{2}=\left[\begin{array}{cccc} \cos\beta & 0 & -\sin\beta & 0\\ 0 & 1 & 0 & 0\\ \sin\beta & 0 & \cos\beta & 0\\ 0 & 0 & 0 & 1 \end{array}\right]$$

### 2.2. Calculation Model for Residual Height of Milling Surface Morphology

**Figure 3:**
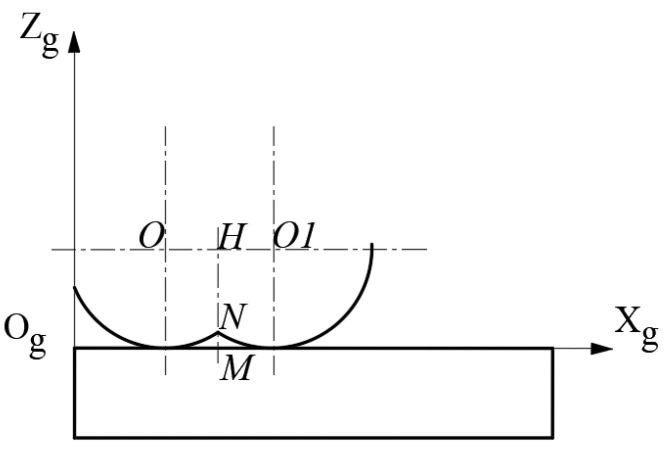
Schematic diagram of residual height of surface morphology.

Figure 3 illustrates a schematic representation of the residual height in the surface morphology. The arcs *O* and *O1* are the surface morphologies formed between adjacent teeth during the milling process, and point *N* is the vertex of the residual height of the surface morphology. As can be seen, the value of residual height ***MN*** is expressed by the following formula:

(6) $$MN=\frac{8z_{0}+\sqrt{64z_{0}^{2}-16(x_{1}^{2}-2x_{1}x_{0}+x_{0}^{2}+4z_{0}^{2}-r^{2})}}{8}$$

### 2.3. Multi Factor Modeling of Ball End Milling Cutter Motion Trajectory

#### 2.3.1. Modeling of the Influence of Tool Vibration on Cutting Edge Motion Trajectory

During milling, tool vibration inevitably alters the cutting trajectory and directly affects the quality of the finished workpiece surface. Therefore, this article assumes that the tool vibrates along the X, Y, and Z directions without interfering with each other, and represents the tool vibration by the displacement of the center of the ball of the ball end milling cutter in the X, Y, and Z directions, in order to construct a tool vibration model. This assumption is based on the following considerations: (1) The stiffness distribution characteristics of the tool structure in the high-speed milling process often lead to the axial vibration to dominate, while the mutual interference effect is relatively weak; (2) This paper focuses on the influence of vibration on the residual height of the surface; (3) Available literature indicates (for example, Sun et al. [24]) that under normal machining parameters, the mutual interference between XYZ vibrations has a limited influence on the surface prediction error. The structural design and dynamic stiffness distribution of high speed milling tools often lead to dominant vibrations in a single direction at any given time, particularly under steady cutting conditions. Therefore, in order to balance the complexity and computational efficiency of the model, the interference of lateral vibration is simplified in this study.

The vibration of the cutting tool is similar to that of a spring damping system, and the dynamic model of the system can be expressed by Equation (7) [24].

(7) $$\begin{array}{c} \frac{d^{2}x}{dt^{2}}+2\xi_{x}w_{x}\frac{dx}{dt}+w_{x}^{2}x\left(t\right)=\frac{w_{x}^{2}}{k_{x}}F_{x}\left(t\right)\\ \frac{d^{2}y}{dt^{2}}+2\xi_{y}w_{y}\frac{dy}{dt}+w_{y}^{2}y\left(t\right)=\frac{w_{y}^{2}}{k_{y}}F_{y}\left(t\right)\\ \frac{d^{2}z}{dt^{2}}+2\xi_{z}w_{z}\frac{dz}{dt}+w_{z}^{2}z\left(t\right)=\frac{w_{z}^{2}}{k_{z}}F_{z}\left(t\right) \end{array}$$

In this formula, ***F_x_***(***t***) represents the cutting force on the *X*-axis; ***F_y_***(***t***) represents the cutting force on the *Y*-axis; ***F_z_***(***t***) represents the cutting force on the *Z*-axis; ***ξ*** is the damping coefficient of the vibration system; ***ω*** is the natural frequency of the vibration system under dynamic conditions; ***k*** is the modal stiffness of the vibration system under dynamic conditions.

By solving the above vibration equation, the coordinate transformation matrix due to tool vibration can be obtained as:

(8) $$M_{3}=\left[\begin{array}{cccc} 1 & 0 & 0 & -x(t)\\ 0 & 1 & 0 & -y(t)\\ 0 & 0 & 1 & -z(t)\\ 0 & 0 & 0 & 1 \end{array}\right]$$

#### 2.3.2. Modeling of the Influence of Tool Eccentricity on Cutting Edge Motion Trajectory

**Figure 4:**
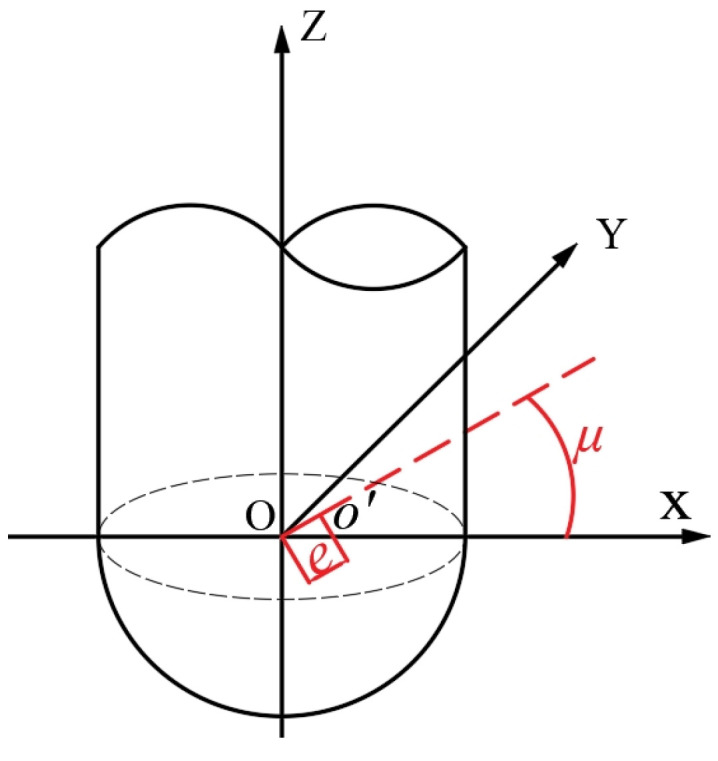
Schematic diagram of eccentricity of ball nose milling cutter.

In order to enhance the accuracy of the simulation, potential misalignment between the tool axis and the spindle axis must be considered in the model (i.e., the eccentricity introduced during tool mounting; see Figure 4).

Within the coordinate frame of the workpiece, let ***e*** be the radius of the eccentric circle caused by spindle rotation, and let ***μ*** be the eccentric angle between the tool and spindle axes. Then, the correction matrix for the tool’s eccentric cutting-edge motion can be expressed as:

(9) $$M_{4}=\left[\begin{array}{cccc} 1 & 0 & 0 & -\mathrm{ecos}\mu \\ 0 & 1 & 0 & -\mathrm{esin}\mu \\ 0 & 0 & 1 & 0\\ 0 & 0 & 0 & 1 \end{array}\right]$$

#### 2.3.3. Modeling of the Influence of Tool Deformation on Cutting Edge Motion Trajectory

In high-speed milling, the tool is subject to vibration and deformation under cutting forces. Let ***x***(***z_j_***,***t***) and ***y***(***z_j_***,***t***) denote the tool’s deformation in the x and y directions, respectively. The deformation of the ball-end mill in the x-direction is given by [25]:

(10) $$x\left(z_{j},t\right)=\frac{F_{x}}{6EI}\left[{\left(r_{x}\left(\theta \right)-z\right)}^{3}-{\left(L-Z\right)}^{3}+3{\left(L-Z\right)}^{2}\left(L-r_{x}\left(\theta \right)\right)\right]$$

In the formula, ***I*** denotes the moment of inertia acting on the ball-end milling cutter; ***E*** represents the elastic modulus of the tool material; ***L*** denotes the length of the ball-end milling cutter; ***F_x_*** representing the component of cutting force in the *x*-direction; ***r_x_***(***θ***) represents the center position of the instantaneous cutting edge along the *x*-axis.

In the direction of the *z*-axis, the surface plastic deformation of the workpiece together with its vibration are the key contributing factors. The plastic strain attains its peak value at the workpiece surface and exhibits a negative exponential distribution. In the direction perpendicular to the workpiece surface, this value rapidly decreases. The plastic strain [26] can be obtained as follows:

(11) $$\begin{array}{c} \varepsilon \left(z\right)=J_{1}e^{-J_{2}z}\\ J_{1}=\frac{\cos\left(\alpha_{0}\right)}{\sqrt{3}\sin\varphi \cos\left(\varphi -\alpha_{0}\right)}\\ J_{2}=-\frac{1}{\left(n+1\right)d}\ln\left[1-\frac{(n+1)\left(F_{x}\cos\varphi -F_{y}\sin\varphi {\left(\sqrt{3}\sin\varphi \right)}^{n+1}\cos^{n}\left(\varphi -\alpha_{0}\right)\right)}{Ka_{e}a_{p}\cos^{n}\alpha_{0}}\right] \end{array}$$

In the formula, ***α*_0_** represents the rake angle of the tool; ***n*** represents the hardening index; ***K*** represents the yield strength; ***d*** indicates the depth of deformation; ***a_e_*** represents the line spacing, and ***a_p_*** denotes the cutting depth.

The plastic deformation can be obtained from Equations (12) and (13):

(12) $$\delta =\int_{0}^{d}J_{1}e^{-J_{2}z}dx$$

(13) $$\delta_{d}=-\frac{J_{1}}{J_{2}}e^{-J_{2}d}$$

Therefore, the matrix used to correct the influence of tool shape changes on tool paths can be expressed as:

(14) $$M_{5}=\left[\begin{array}{cccc} 1 & 0 & 0 & u\left(z_{j},t\right)+x(t)\\ 0 & 1 & 0 & v(z_{j},t)+y(t)\\ 0 & 0 & 1 & \delta_{d}+z\left(t\right)\\ 0 & 0 & 0 & 1 \end{array}\right]$$

After establishing the geometric model of the spherical cutting edge and calculating the transformation matrices, the motion path equation for any point on the tool’s cutting edge can be described by the following formula:

(15) $$\left[\begin{array}{c} x\prime \\ y\prime \\ z\prime \\ 1 \end{array}\right]=M_{5}M_{4}M_{3}M_{2}M_{1}\left[\begin{array}{c} x\\ y\\ z\\ 1 \end{array}\right]$$

### 2.4. Modeling Method for Surface Morphology

**Figure 5:**
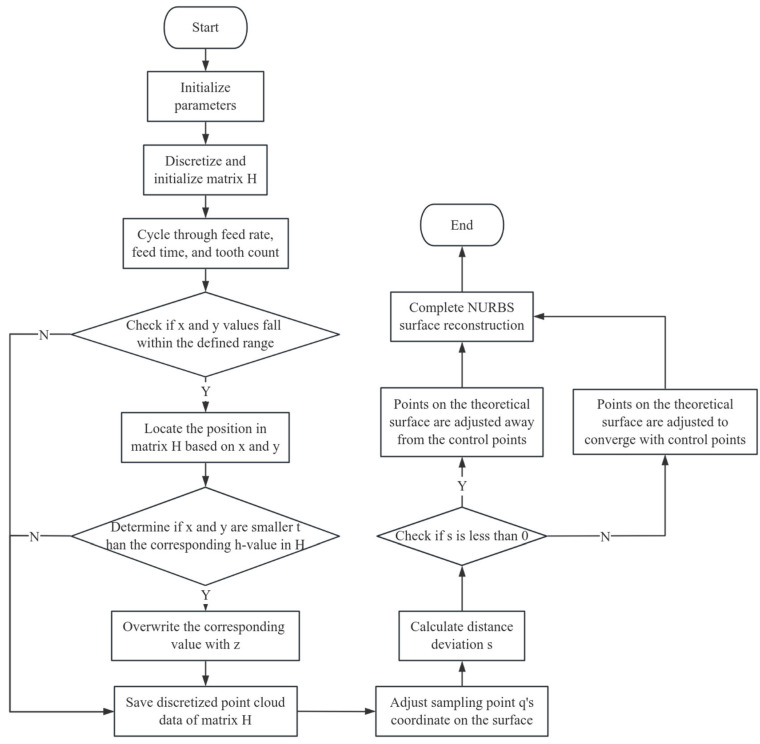
Matlab simulation flowchart of surface morphology.

Given that the surfaces of most milled workpieces belong to free-form surfaces, describing free-form surfaces can be cumbersome. In mechanical manufacturing, there are three primary mathematical representations for free-form surfaces: Bézier, Coons, and NURBS for free-form surfaces: Bezier, Coons, and NURBS (Non-Uniform Rational B-Spline) surfaces. B-spline surfaces have many advantages, especially their ability to locally adjust when depicting complex shape related parts, making NURBS surfaces an ideal choice. Therefore, this article decides to adopt NURBS surface reconstruction based on flexibility to achieve discrete point fitting and obtain surface morphology. The specific steps of this process are shown in Figure 5.

In order to realize the accurate reconstruction of the machining surface, this paper adopts the non-uniform rational B-spline (Non-Uniform Rational B-Splines, NURBS) surface fitting method and reconstructs the workpiece surface morphology by combining the tool trajectory data obtained from simulation. The specific implementation process mainly includes the following four stages: (1) Parameter initialization. Set the basic cutting parameters including feed rate, number of teeth and cutting time, establish the spatial domain of the machining area, and initialize the height matrix H to record the residual height value corresponding to the tool trajectory at each position. (2) Perform trajectory discretization and point cloud generation. By traversing the feed path and time steps, calculate the spatial coordinates of the tool at each moment; determine whether the current coordinate (x, y) falls within the predefined spatial domain. If the condition is met, locate the corresponding position in the height matrix H and compare the calculated height value z with the original value at that position. If the z value is smaller (indicating material removal), update the matrix H and save the point to the point cloud dataset. (3) Perform NURBS fitting and error correction on the generated point cloud. Input the point cloud data into the NURBS surface reconstruction model and calculate the distance deviation s between each sample point and the initial control surface. If s < 0, it indicates that the point is below the theoretical surface, and it needs to be expanded outward along the normal; if s > 0, iterate in the direction of the control points until the error meets the convergence criteria. (4) When all the sampling points converge, the NURBS reconstruction process of surface processing simulation is completed, and the final three-dimensional surface morphology simulation results are obtained.

Compared with traditional Bezier surface, NURBS fitting has higher local control and modeling flexibility, which can better adapt to the processing simulation requirements of complex surface structure.

### 2.5. Materials and Experiments

**Figure 6:**
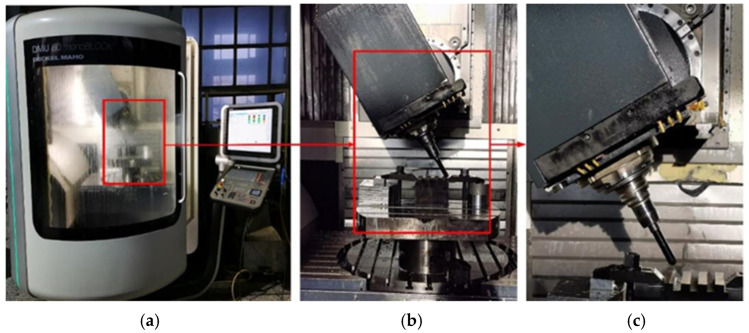
Numerical control machine tool used in the experiment. (**a**) External view of the five-axis CNC milling machine. (**b**) Processing state with 45 ° inclination of main shaft. (**c**) Local enlarged drawing of surface contact.

**Figure 7:**
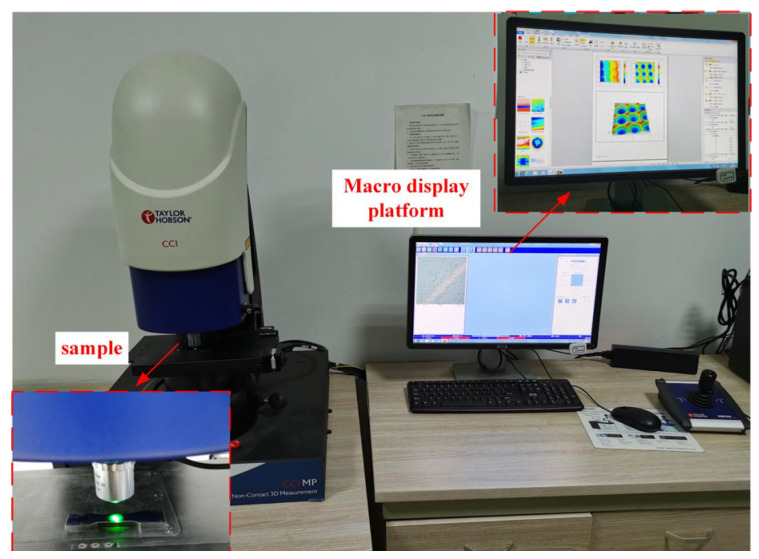
White light interferometer.

The machine tool used in this experiment is the DMU 60mono BLOCK five axis (DMG MORI company, Pfronten, Bavaria, Germany) high-speed vertical machining center, with a spindle speed of up to 10,000 r/min, as shown in Figure 6. The cutting tool used is a SH300-H solid carbide ball-end mill (Xiamen Jinlu, Xiamen, China). A white light interferometer (Figure 7) was used for surface measurement, and the workpiece material was Cr12MoV quenched steel (Daye Special Steel Co., Ltd., Daye, Hubei, China) (70 HRC). See Table 1 for main material parameters.

## 3. Simulation Analysis of the Influence of Processing Parameters on Milling Surface Morphology

In order to study the influence of different process parameters on milling surface morphology, single-factor simulation and experimental research are carried out in this section. In order to ensure the scientific and engineering relevance of the experimental design, based on the available literature, the milling parameters selected in this study under the same processing conditions are: line pitch (*a_e_*: 0.4–0.7 mm), feed per tooth (*f_z_*: 0.4–0.8 mm/z), and cutting depth (*a_p_*: 0.3–0.6 mm), as shown in Table 2, ensuring the representativeness of the experimental results when milling Cr12MoV die steel at high speed.

### 3.1. Simulation Study on the Influence of Line Spacing on Surface Morphology

**Figure 8:**
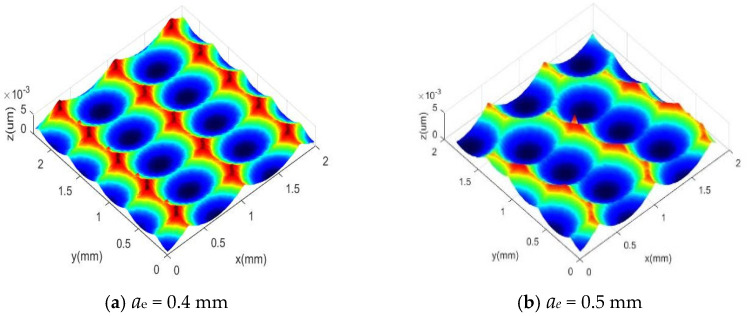

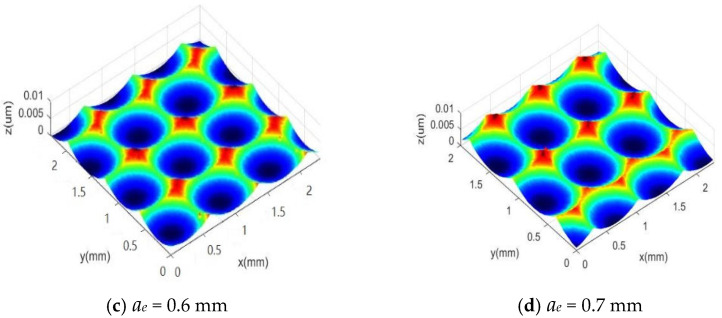
The effect of line spacing on surface morphology (red indicates peaks, and blue indicates valleys).

In traditional milling processes, due to the large line spacing, machining residues are mainly line spacing residues. When milling mold steel with ball end milling cutters, different line spacing can obtain different surface morphologies. Therefore, this study first analyzed the effect of line spacing *a_e_* on the surface morphology in simulation. To further explore the influence mechanism and variation trend of line spacing *a_e_* on surface morphology, a single factor simulation design scheme for line spacing was carried out as shown in Table 2. The tool was fed unidirectionally with a fixed inclination angle of 30°, spindle speed n = 1000 (r/min), line spacing *a_e_*: 0.4~0.7 mm, feed per tooth *f_z_* = 0.4 mm/z, cutting depth *a_p_* = 0.3 mm, Figure 8a–d illustrates the simulated surface morphologies under these conditions.

From the simulation results of Figure 8a–d, it can be seen that when other cutting parameters remain constant and only the milling line spacing a_e_ changes, the surface morphology is periodically and uniformly distributed along the line spacing direction and feed direction. However, the change in line spacing a_e_ only affects the surface morphology in the line spacing direction. With the continuous increase of line spacing *a_e_*, the surface morphology undergoes significant changes in the line spacing direction. The length dimension of the micro unit in the line spacing direction gradually increases with the continuous increase of line spacing a_e_, and the frequency of residual height in the line spacing decreases with the increase of line spacing a_e_. The value of residual height gradually increases, while the length in the feed direction remains basically unchanged.

### 3.2. Simulation Study on the Influence of Feed per Tooth on Surface Morphology

**Figure 9:**
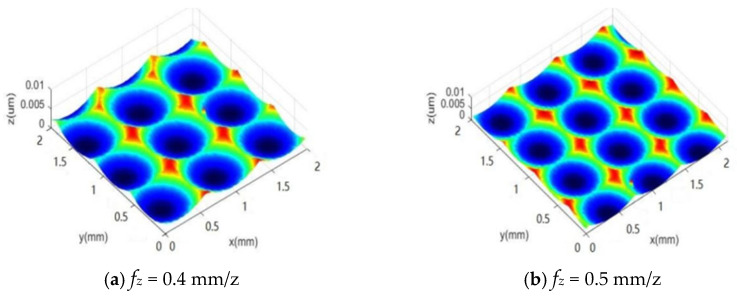

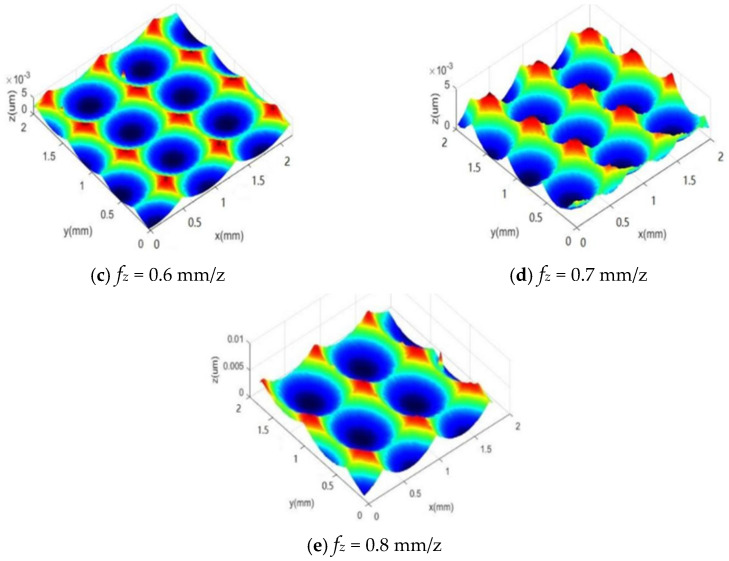
Effect of changing the feed per tooth on the surface topography (red indicates peaks, and blue indicates valleys).

According to the simulation analysis, it is evident that variations in feed per tooth lead to distinct differences in the resulting surface morphology. During the ball-end milling process, the feed per tooth *f_z_* is a key factor influencing the surface characteristics. To explore how the feed per tooth *f_z_* affects surface formation, a single-factor simulation was performed (see Table 2), line spacing *a_e_*: 0.4 mm, feed per tooth *f_z_* = 0.4~0.8 mm/z, cutting depth *a_p_* = 0.3 mm, Figure 9a–e show the simulated surface morphologies under these feed rates.

The simulation results of surface morphology shown in Figure 9a–e indicate that the surface presents a periodic and uniform distribution pattern in both the feed direction and the line spacing direction. Nevertheless, there are slight differences in the variation law. In contrast to the influence of line spacing on surface contour, the feed per tooth *f_z_* primarily influences the surface profile along the feed direction. With the increase of feed per tooth *f_z_*, the length of the microstructure along the feed direction grows continuously, the frequency of residual height decreases, and the residual height value gradually rises.

### 3.3. Simulation Study on the Influence of Cutting Depth on Surface Morphology

**Figure 10:**
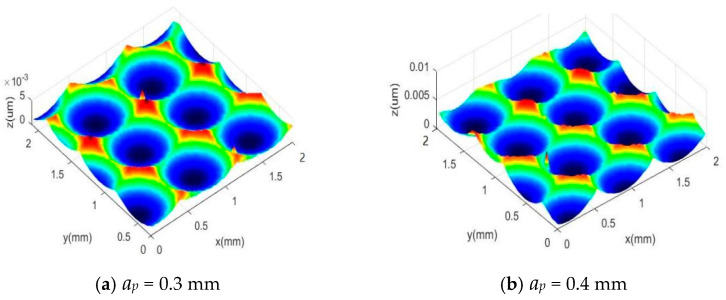

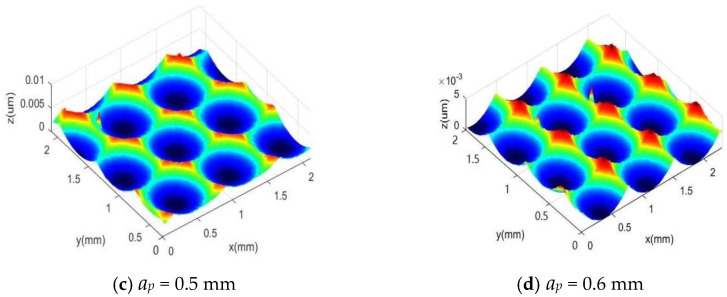
The effect of cutting depth on surface morphology (red indicates peaks, and blue indicates valleys).

Variations in cutting depth during the milling process have a significant impact on the surface morphology. To investigate how milling depth influences surface characteristics and their changing patterns, this study carried out a single-factor simulation scheme focusing on the variation of cutting depth *a_p_*. The specific data can be observed in Table 2, with line spacing *a_e_*: 0.4 mm, feed per tooth *f_z_* = 0.6 mm/z, and cutting depth *a_p_* = 0.3~0.6 mm. Figure 10a–d shows the simulation results of the workpiece surface morphology.

As shown in the simulation results in Figure 10, it is evident that the length of microstructural units on the machined surface slightly increases with the rise of cutting depth *a_p_*, although this increase is limited. The reason for this phenomenon is that a higher cutting depth *a_p_* causes an enlargement of the milling radius of the ball-end mill engaged in the workpiece, thereby contributing to the growth of the microstructure length. However, since the radius of the cutter’s ball end is significantly larger than the cutting depth *a_p_*, the overall increase in microstructure length remains relatively small. Additionally, as the cutting depth *a_p_* continues to rise, the residual height generated by the tool path interval and feed direction also increases correspondingly, resulting in a continuous growth of surface roughness values.

### 3.4. Simulation Study on the Influence of Phase Difference on Surface Morphology

**Figure 11:**
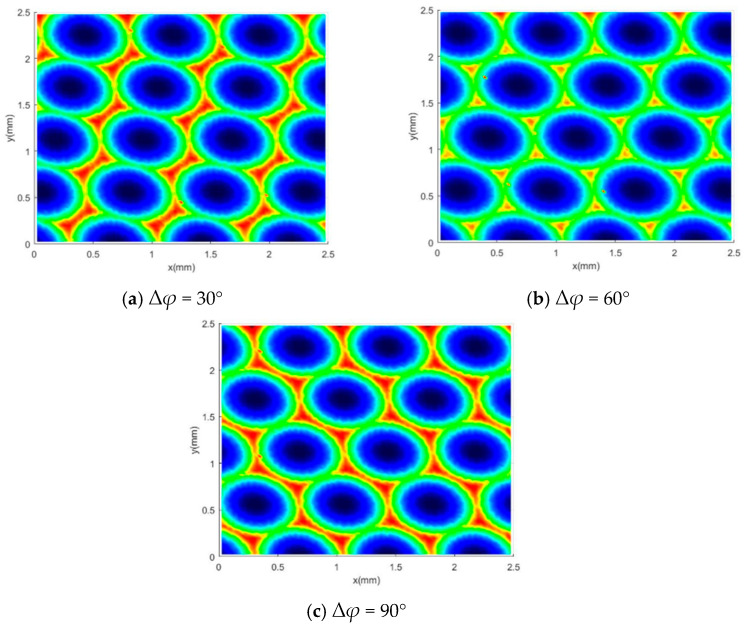
The effect of phase difference Δ*φ* on surface morphology (red indicates peaks, and blue indicates valleys).

During high-speed ball-end milling, the phase difference Δ*φ* between adjacent cutting edges plays a crucial role in determining the formation of the workpiece surface microstructure. Traditional simulation models often overlook the relative angular relationship between cutting teeth. In this work, a surface morphology prediction model is established that accounts for the overlapping offsets of adjacent cutter trajectories, which significantly enhances simulation accuracy. This model is used to explore the effects of different initial phase angles *φ*_0_ on cutting trajectory interference behavior and surface residual height (Figure 11).

The figure shows the simulated morphology of ball head milling when Δ*φ* is randomly set to 0°, 30°, 60°, and 90°. It can be observed that (1) when the phase difference is a certain value, the surface texture direction is always the same, and the surface morphology is arranged in a regular pattern; (2) when the phase difference is random (i.e., the cutting edges are out of phase by a random angle), the surface morphology and texture arrangement become chaotic, with no regular pattern, and no regular texture arrangement is formed; (3) when the phase difference changes from 0° to 90°, the surface morphology gradually changes from a quadrilateral to a hexagon, and the direction angle of the surface texture gradually increases with the increase of the phase difference; (4) when the phase difference is 0°, the surface morphology is a regular quadrilateral morphology, and when the phase difference is 90°, the surface morphology is a regular hexagonal pattern.

## 4. Experimental Study on Surface Morphology of High-Speed Ball Head Milling Processing

### 4.1. Experimental Study on the Influence of Line Spacing on Surface Morphology

**Figure 12:**
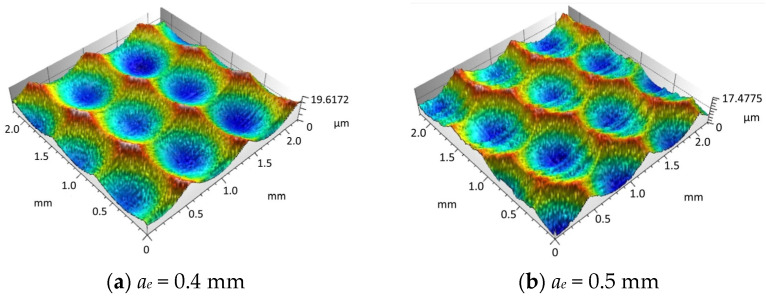

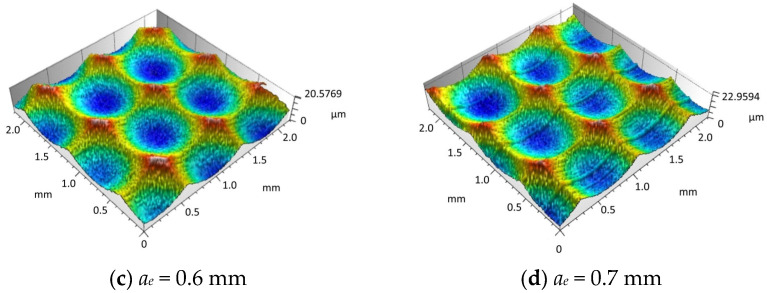
Experimental results of the influence of line spacing on surface morphology.

In order to validate the accuracy of the simulation results regarding the effect of line spacing on surface morphology, this paper designed a single factor experiment on milling line spacing. Firstly, the samples were machined under the specified conditions, and then the surface morphologies of different experimental samples with milling line spacing were observed under a microscope, as illustrated in Figure 12a–d.

**Figure 13:**
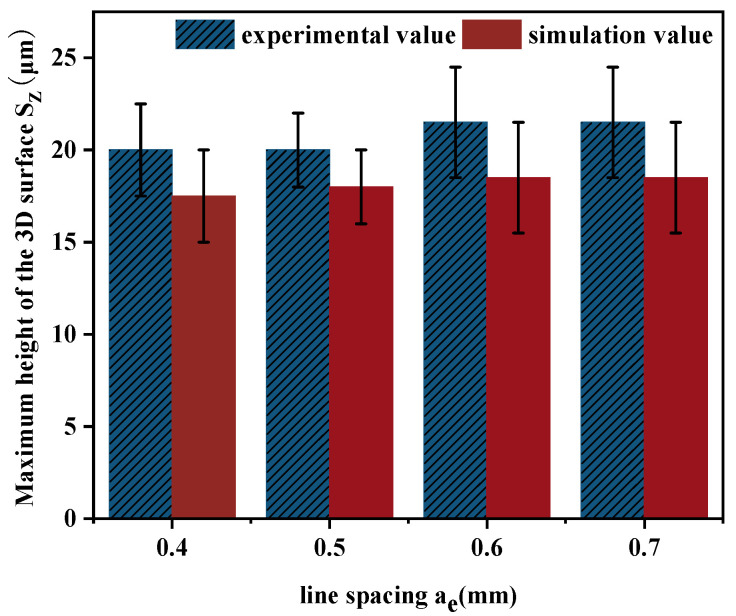
Comparison of surface roughness between different *a_e_* experiments and simulations.

Through the comparison and analysis of the experimental data and the simulation results, it can be concluded that the simulated milling surface morphology is relatively consistent with the experimental findings. Figure 13 presents the variation trend of the three-dimensional surface roughness by comparing the experimental measurements with the simulated values.

As illustrated in Figure 13, the simulated surface roughness values of the workpiece show a high degree of consistency with the experimental measurements. With the continuous increase in line spacing *a_e_*, the surface roughness values exhibit a gradual upward trend. This phenomenon is mainly attributed to the fact that a larger line spacing expands the distance between adjacent tool paths, resulting in more uncut material remaining on the workpiece surface. Consequently, the residual height in the line spacing direction increases, causing the overall three-dimensional surface roughness to rise. The deviation between the simulation results and experimental data is within the range of 7% to 15%, and the simulated values are generally smaller than the corresponding experimental values.

### 4.2. Effect of Feed per Tooth on Surface Morphology

**Figure 14:**
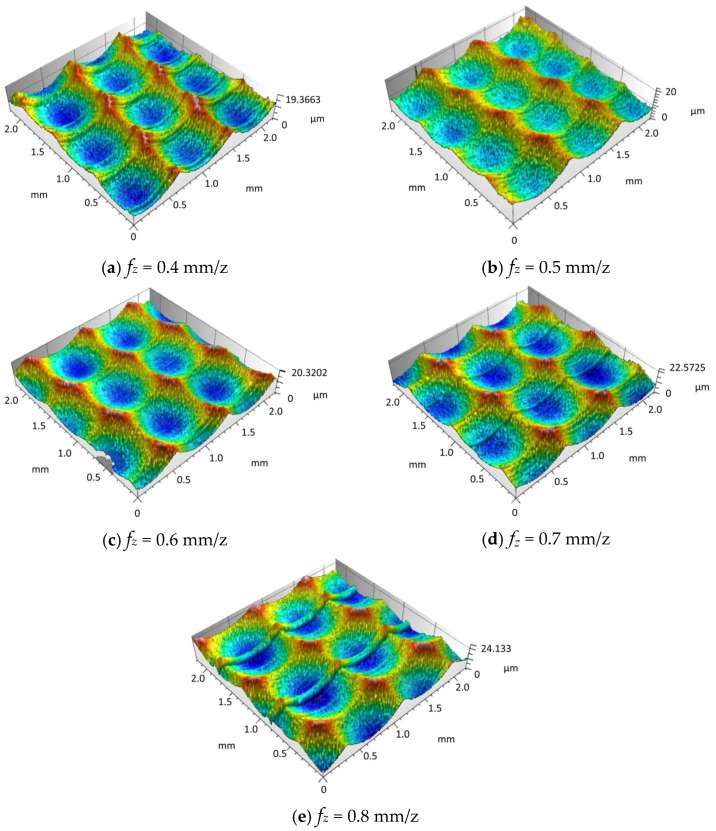
Experimental results of the influence of feed per tooth on surface morphology.

Based on simulation experiments, it is known that the difference in feed per tooth will result in different surface morphologies. To validate the simulation results for feed per tooth, we conducted a single-factor experiment varying the feed per tooth on surface morphology, this paper designed a single factor experiment on feed per tooth. Firstly, the samples were processed for morphology, and then the surface morphology of different experimental samples was observed microscopically to obtain the measured surface morphology of samples with different feed per tooth rates, as shown in Figure 14a–e.

**Figure 15:**
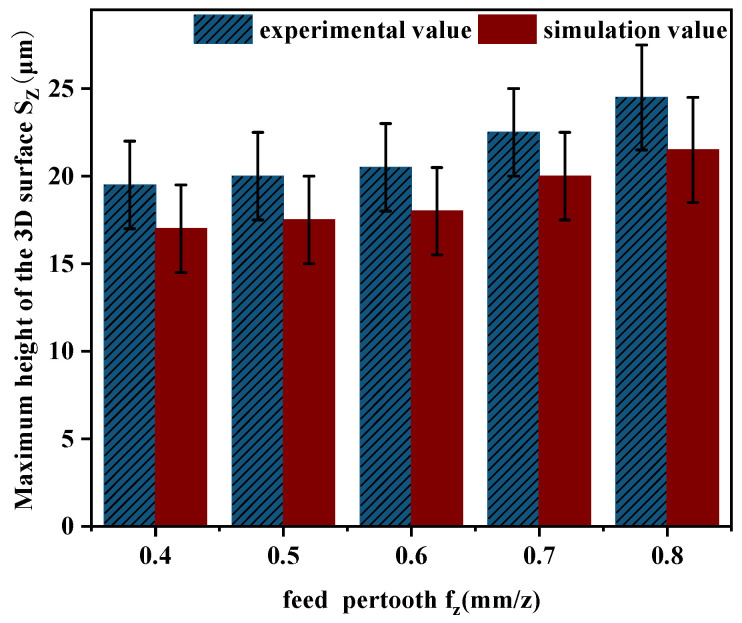
Comparison of surface roughness between fz experiment and simulation.

Through the comparison and analysis of the experimental data obtained from actual machining and the simulation results generated by MATLAB software, it is evident that the simulated surface morphology is relatively consistent with the experimental findings. Figure 15 shows the comparison of the 3D surface roughness obtained from experimental measurements and simulation results.

As illustrated in Figure 15, the comparison between the experimental results of surface roughness *Sz* and the simulated data shows that both exhibit a consistent variation pattern. In particular, the surface roughness increases progressively as the feed per tooth fz rises. This behavior is mainly caused by the fact that a larger feed per tooth fz produces a higher residual height along the feed direction, thereby amplifying the surface roughness. However, there remains an error between the experimental and simulated results, ranging from approximately 7% to 15%, primarily due to the increase in cutting force generated by a higher feed per tooth fz during machining. In actual machining, surface morphology with varying depths and sizes is further formed due to factors such as tool deformation and cutting vibration. However, in the simulation cutting process, it is a relatively stable and ideal environment, and the resulting surface morphology is also relatively stable and regular, resulting in certain errors be-tween simulation values and experimental values.

### 4.3. The Influence of Cutting Depth on Surface Morphology

**Figure 16:**
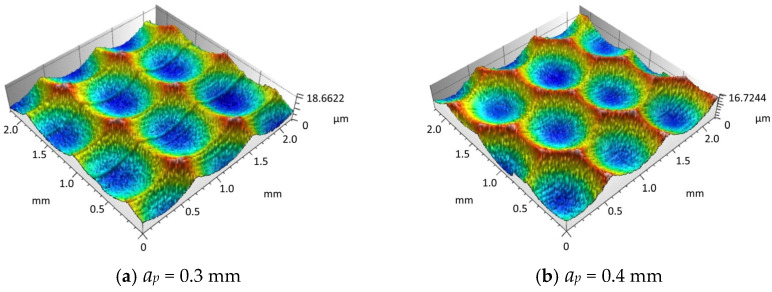

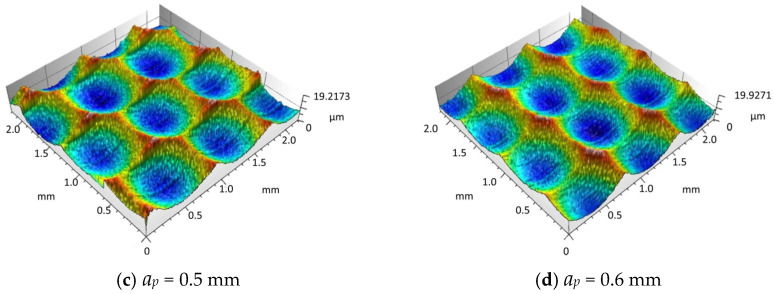
Experimental results of the influence of cutting depth on surface morphology.

To validate the accuracy of the simulation results regarding the effect of cutting depth on surface morphology, this paper designed a single factor experiment on cutting depth. Firstly, the samples were processed for morphology, and then the surface morphology of different experimental samples was observed microscopically. As shown in Figure 16a–d, the measured surface morphology corresponds to samples processed with different cutting depths.

**Figure 17:**
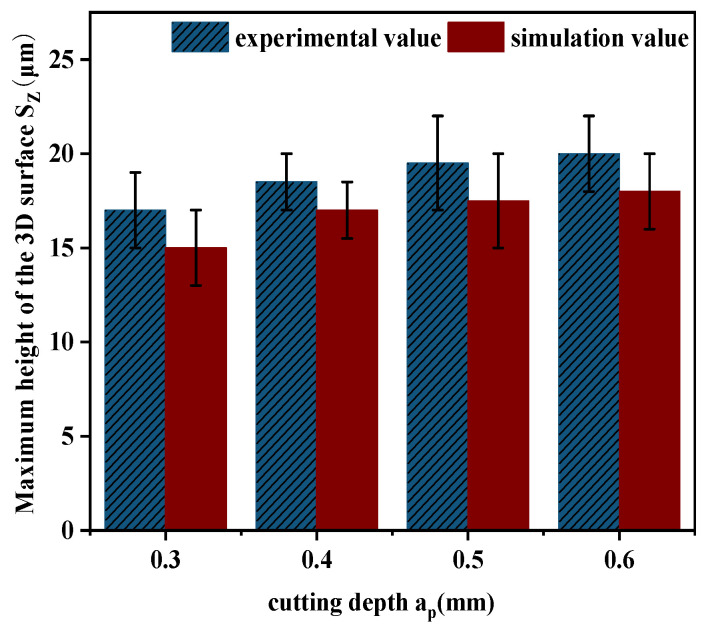
Comparison of surface roughness between *a_p_* experiments and simulations.

Through the comparative analysis of the experimental results obtained from actual machining and the simulation results generated by MATLAB software, it is observed that the simulated surface morphology is relatively consistent with the experimental findings. The corresponding comparison of the experimental and simulated values of the three-dimensional surface roughness is illustrated in Figure 17.

Figure 17 presents the comparison of the measured and simulated values of the three-dimensional surface roughness *Sz* corresponding to different cutting depths. It is observed that with the increase of cutting depth *a_p_*, both experimental and simulated surface roughness values rise accordingly. This is mainly due to the fact that a larger cutting depth results in a higher radial cutting force, which strengthens the tool’s motion and vibration, thus causing the surface roughness to increase. Consequently, there is a certain deviation between the simulation and experimental results, ranging from 7% to 15%.

Haining Gao et al. [14] evaluated quenched Cr12MoV steel and found that the maximum deviation between simulated and experimental roughness reached 21.9%, while the maximum deviation in this study was 15%, which is within a controllable range and has good reference value. The main reason for the differences lies in the different experimental equipment used. Three axis CNC machine tools were used, while a five axis high-speed vertical machining center was used in this study. The spindle stiffness of the machine tool was better, the cutting speed was faster, and the maximum spindle speed could reach 18,000 r/min. The vibration was smaller, and the milling process was more stable and conducive to forming a regular surface morphology. The influence of tool wear was not considered (in order to reduce the impact of tool wear on this study, new blades were replaced after each milling). Therefore, the roughness deviation in this study is relatively small.

### 4.4. The Influence of Phase Angle on Surface Morphology

To further verify the actual effect of phase angle on the surface morphology of ball head milling, this paper designed high-speed milling experiments under different tooth to tooth phase difference conditions. Selecting random angles of 0°, 30°, 60°, and 90° with Δ*φ* as the research objects, ensuring that other cutting parameters (feed rate, line spacing, cutting depth, etc.) remain consistent.

**Figure 18:**
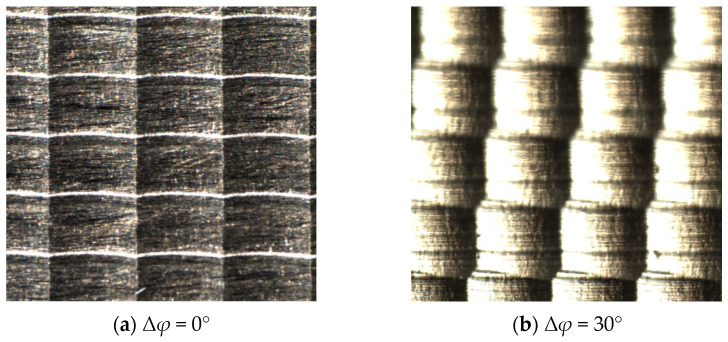

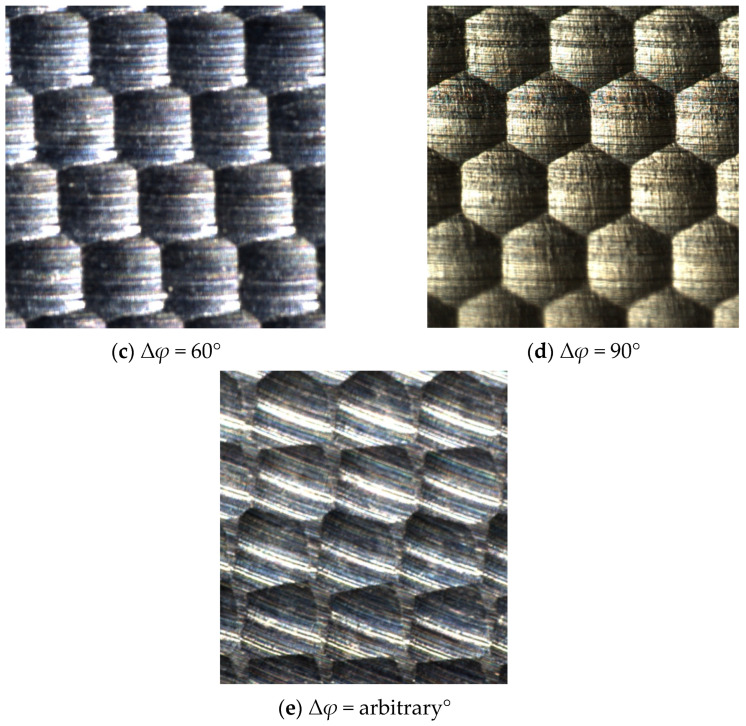
Experimental results of the influence of phase difference Δ*φ* on surface morphology (Δ*φ*).

According to the high-speed ball head milling experiments shown in Figure 18a–e, By comparing and analyzing the experimental data with the simulation outcomes, it is evident that the simulated surface morphology in the milling process is relatively consistent with the experimental observations. From Figure 18, it can be seen that when the phase difference remains constant, the surface texture direction is always the same, and the surface morphology is arranged in a regular pattern. When the phase difference randomly changes, the arrangement of morphology and texture becomes chaotic. Due to the fact that the number of cutting edges of the ball end milling cutter is 2, the morphology gradually changes from a quadrilateral to a hexagon when the phase difference is from 0~90°, and the texture direction gradually increases. When the phase difference is 0, the surface morphology is a regular quadrilateral morphology, and when the phase difference is 90°, the surface morphology is a regular hexagonal morphology.

## 5. Conclusions

This study proposes a ball-end milling surface morphology prediction model that considers factors, such as tool vibration, eccentricity, and deformation. Based on the theoretical model, the effects of different machining parameters (such as feed rate, line spacing, cutting depth, and phase angle) on surface morphology and roughness were investigated. A validation milling experiment was conducted on quenched steel Cr12MoV workpieces. The main results and conclusions are summarized as follows:

A simulation model based on the Z-map algorithm and MATLAB was developed, and the surface morphology of quenched steel was simulated and analyzed, revealing the formation mechanism of the surface morphology. The simulation results showed good consistency with experimental values. The deviation in three-dimensional surface roughness remained within the range of 7% to 15%, confirming the model’s accuracy and reliability. The main reason for the residual deviation is that the simulation assumes ideal conditions without considering factors such as tool axial movement or vibration. In addition, in the actual cutting process, there is a small gap between the tool and the workpiece, which may cause surface scratch and material smearing and increase roughness. Furthermore, tool wear and the rigidity of the machine tool spindle also affect the surface roughness. The combined effects of these factors will lead to surface morphology errors between the simulation and experimental values.

Different machining parameters have a significant impact on the surface morphology of the workpiece. The line spacing (*a_e_*) and feed per tooth (*f_z_*) significantly affect the surface residual height. As the line spacing and feed per tooth increase, the surface residual height and surface roughness also increase markedly. The cutting depth (*a_p_*) has a relatively minor effect on surface morphology, but as the cutting depth increases, the surface roughness also tends to rise. Through simulation analysis, it is observed that the surface morphology of machining presents periodic distribution in the feed path and the interval direction of adjacent tool trajectory.

The phase difference between cutting edges plays a crucial role in influencing the surface morphology of machined surfaces. When the phase difference is at a specific value, the surface texture direction remains consistent, resulting in regular and periodic surface patterns; introducing the phase angle makes the model’s predictions closer to actual measurements, providing a theoretical basis and design reference for tool structure optimization and high-quality surface control.

## Figures and Tables

**Table 1:** Chemical composition of Cr12MoV [27].

Element	C	Cr	Mo	V	Mn
content	1.45–1.70	11.00–12.50	0.4–0.6	0.15~0.30	≤0.40
element	Si	Ni	Cu	S	P
content	≤0.40	≤0.25	≤0.30	≤0.030	≤0.030

**Table 2:** Single-factor simulation and experimental scheme of different milling parameters.

Serial Number	Line Spacing *a_e_* (mm)	Feed per Tooth *f_z_* (mm/z)	Cutting Depth *a_p_* (mm)	Spindle Speed *n* (r/min)
a	0.4	0.4	0.3	10,000
b	0.5	0.5	0.4	
c	0.6	0.6	0.5	
d	0.7	0.7	0.6	
e	-	0.8	-	

## Data Availability

The original contributions presented in this study are included in the article. Further inquiries can be directed to the corresponding author.

## Nomenclature

The key parameters and symbols used in this study are listed below:

*a_e_*	line spacing (radial distance between adjacent cutter paths)
*a_p_*	cutting depth (axial depth of material removal)
*f_z_*	feed per tooth (distance advanced per cutter tooth)
phase difference (Δ*φ*)	phase difference between adjacent cutter teeth (in degrees)
residual height	uncut material height between adjacent scallop tracks
